# Prevalence of Workplace Violence Types against Personnel of Emergency Medical Services in Iran: A Systematic Review and Meta-Analysis

**Published:** 2019-10

**Authors:** Ali Sahebi, Katayoun Jahangiri, Sanaz Sohrabizadeh, Mohamad Golitaleb

**Affiliations:** 1 Department of Health in Disasters and Emergencies, School of Public Health and Safety, Shahid Beheshti University of Medical Sciences, Tehran, Iran.; 2 Safety Promotion and Injury Prevention Research Center, Shahid Beheshti University of Medical Sciences, Tehran, Iran.; 3 Department of Nursing, School of Nursing and Midwifery, Arak University of Medical Sciences, Arak, Iran.

**Keywords:** *Emergency Medical Services (EMS)*, *Systematic Review and Meta-Analysis*, *Workplace Violence*

## Abstract

**Objective:** Workplace violence is one type of occupational hazards that is increasingly growing worldwide. In the health system, one of the important groups subject to workplace violence is emergency medical services (EMS) personnel, who provide emergency services for patients and casualties as the first responders. The aim of this study was to determine the prevalence of workplace violence and its different types among Iranian EMS personnel.

**Method** **:** This study was conducted based on PRISMA guideline for systematic review and meta-analysis. The data were extracted from Scopus, PubMed, Web of Science, Google Scholar, SID and MagIran databases using Persian and English keywords. The search was conducted up to December 2018 without any limitation in publication year. The qualities of selected papers were assessed by STROBE checklist. I^2^ index was used to evaluate heterogeneity, and random effects model was used in meta-analysis. Data were analyzed using Stata14.

**Results: **A total of 9 studies entered the meta-analysis. The total sample size was 1257 Iranian EMS personnel, with an average age of 32.21 ± 2.01 years. The prevalence of physical, verbal, and cultural workplace violence among EMS personnel was 36.39% (CI 95%:27.29–45.50, P<0.001, I^2^ = 90.8%), 73.13% (95% CI=68.64-77.62, P=0.013, I^2^ = 62.7%), and 16.51% (95% CI =3.49- 29.53, p<0.001. I^2^ = 94.7%), respectively.

**Conclusion: **Considering the high prevalence of workplace violence among EMS personnel in Iran, more studies should be conducted to determine the underlying causes of EMS staff workplace violence in Iran. Training violence prevention methods as well as assigning national protective rules are highly suggested. Insufficient studies on Workplace violence among EMS personnel in Iran and high heterogeneity were the limitations of this study.

Violence is defined as an intentional use of physical strength, or as a threat or action against itself, someone else, a group, or community (1). According to the definition by the World Health Organization, violence includes physical assault, murder, verbal abuse, bullying/mobbing, sexual and racial harassment, and psychological stress (2). Workplace violence (WPV) is an increasingly-growing health problem and the common cause of health disturbance in emergency medical services (EMS) personnel around the world. WVP is classified in to physical, verbal, and cultural violence (3-6). Violence has a negative effect on the health of EMS personnel (7). 

WPV is considered as one of the important factors leading to termination of employment and job dissatisfaction in medical personnel and has a large effect on the quality of services, patients’ satisfaction, and efficacy and efficiency of personnel performance. An overview of databases shows that WPV in medical centers is not specific to a certain community or setting (8). Also, 70%-80% of physicians, nurses, and EMS personnel experience one or more instances of violence yearly (9). The results of the study of Alharthy et al showed that EMS personnel experienced 65% WPW, of which 61% were verbal WPV (10). 

The rate of WPW among EMS personnel was reported higher than firefighters (11).

The study of the Rafeea et al showed that physical, verbal, and sexual WPW in emergency department staff were reported as 11%, 78%, and 3% respectively (12). EMS personnel, as a critical component of health care, public health, and disaster response systems, encounter patients who need emergency services. They meet various patients in different situations and settings. They may have a patient in the scene of an accident, a public place, on a street, or even in a forest and have to provide medical services. Therefore, these personnel cannot avoid working in stressful situations and should provide immediate medical care and medical first aids at the scene of accidents and on the way back to the hospital. Provision of appropriate prehospital services requires effective communication between EMS personnel and patients and their companions. EMS personnel are at the risk of violence by the patient or their companions due to nature of their occupation. Sometimes, it has been observed or heard that there is a conflict between ambulance technicians and patients or their companions (3-13-14). The present study was conducted to estimate the prevalence of workplace violence among Iranian EMS personnel by a systematic review and meta-analysis.

## Materials and Methods

***Search strategy***

In this study, the physical, verbal, and cultural WPV against Iranian EMS personnel was reviewed based on the studies published in the national and international databases, such as SID, MagIran, Google Scholar, PubMed, Web of Science, and Scopus without time limitations until December 2018. The search was conducted using both Persian and English approved keywords including violence, aggression, assault, verbal, physical, cultural, workplace, at work, occupational, ambulance, paramedic, prehospital emergency staff, prehospital emergency personnel, emergency medical services, Emergency Medicine Technicians, and Iran. A combination of AND/OR operators was used in the searches. Manual searches were conducted to identify other records. For example, the search strategy in the PubMed database was as follows: ((("workplace violence"[MeSH Terms] OR ("workplace"[All Fields] AND "violence"[All Fields]) OR "workplace violence"[All Fields]) AND ("emergency medical services"[MeSH Terms] OR ("emergency"[All Fields] AND "medical"[All Fields] AND "services"[All Fields]) OR "emergency medical services"[All Fields])) OR (prehospital[All Fields] AND ("emergencies"[MeSH Terms] OR "emergencies"[All Fields] OR "emergency"[All Fields]))) AND ("Iran"[MeSH Terms] OR "Iran"[All Fields]).

***Study Selection***

This study was conducted based on PRISMA (Preferred Reporting Items for Systematic Reviews and Meta-analyses) checklist (15). Initially, all the relevant studies containing data on the prevalence of physical, verbal, and cultural WPV against EMS personnel were extracted. The studies were selected based on inclusion and exclusion criteria. The exclusion criteria were unrelated studies, case reports, letters to editor, systematic reviews, interventional studies, duplicate publication, WPV in other health care groups or students, not reporting the prevalence types of WPV, and lack of access to the full-text of the articles. The researchers evaluated the selected studies using STROBE checklist (16), which contains 22 items and evaluates various aspects. Each question in the checklist can be scored from zero to two, and the minimum and maximum checklist scores are 0 and 44, respectively. The selected studies were categorized into three groups of low (0 - 15 scores), medium (16 - 30 scores), and high (31 - 44 scores) quality. Finally, all the relevant studies acquiring a minimum score of 16 entered the meta-analysis phase.

***Data Extraction***

In each stage, two researchers independently conducted the search, study selection, quality evaluation, and data extraction to prevent bias. Disagreement between the two researchers was resolved by a discussion with a third researcher. The researchers used a prepared checklist to extract the data, which included the study’s first author, year, place, sample size, sampling method, study design, participants’ average age, and the prevalence of physical, verbal, and cultural WPV.

***Statistical Analysis***

Binomial distribution was used to calculate the variance of each study and the weighted average was used to combine the prevalence rates reported in different studies. Each study was weighed in the reverse proportion to its variance. I^2^ index was used to evaluate heterogeneity of the studies. The heterogeneity of physical, verbal, and cultural WPV was calculated as 90.8%, 62.7%, and 94.7%, respectively, which showed that the heterogeneity of physical and cultural WPV lies in the category of high heterogeneity and that of verbal WPV lies in the category of moderate heterogeneity. Heterogeneity has 3 categories of less than 25% (low heterogeneity), 25% to 75% (moderate heterogeneity), and more than 75% (high heterogeneity) (17). Therefore, due to the large difference in the prevalence rates reported in various studies and significance of the heterogeneity index (I^2^), random effects model was used in the meta-analysis. To investigate the relationship between the prevalence of physical, verbal, and cultural WPV with the study’s year, meta-regression was used. Also, Begg’s test was used for evaluating publication bias. Finally, the data were analyzed using STATA software (Version 14).

## Results

**Figure 1:**
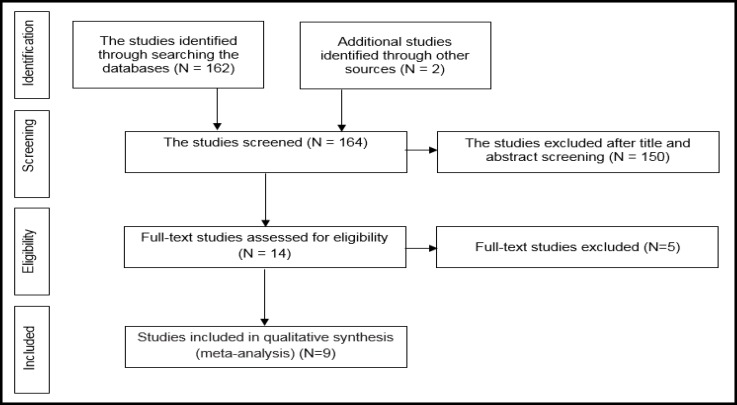
Flowchart of the Study and Selection of Studies Based on PRISMA Steps

**Figure 2:**
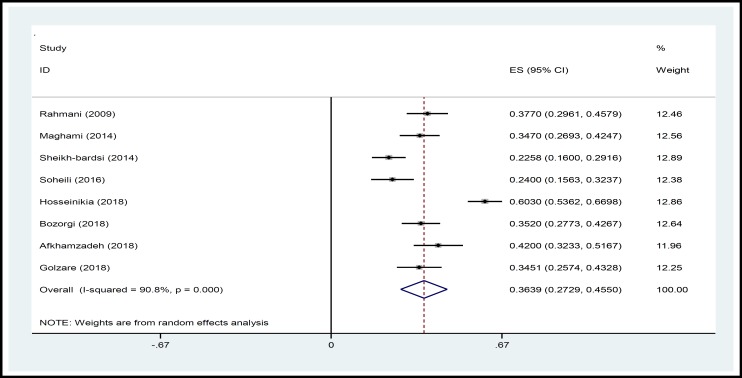
Forest Plot Shows the Prevalence Rate of Physical WPV in Overall and Separately

**Figure 3:**
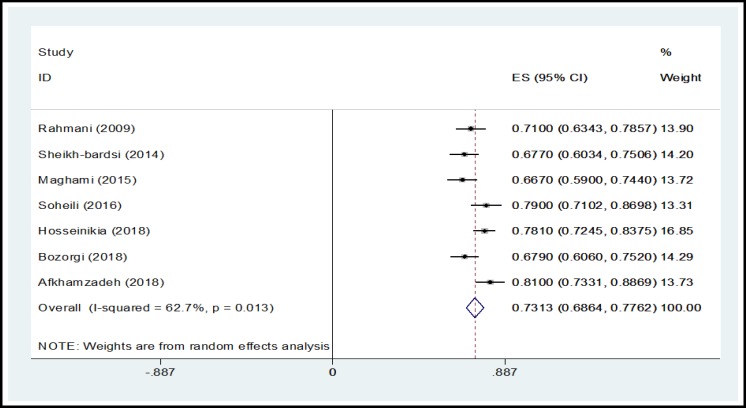
Forest Plot Shows the Prevalence Rate of Verbal WPV in Overall and Separately

**Figure 4:**
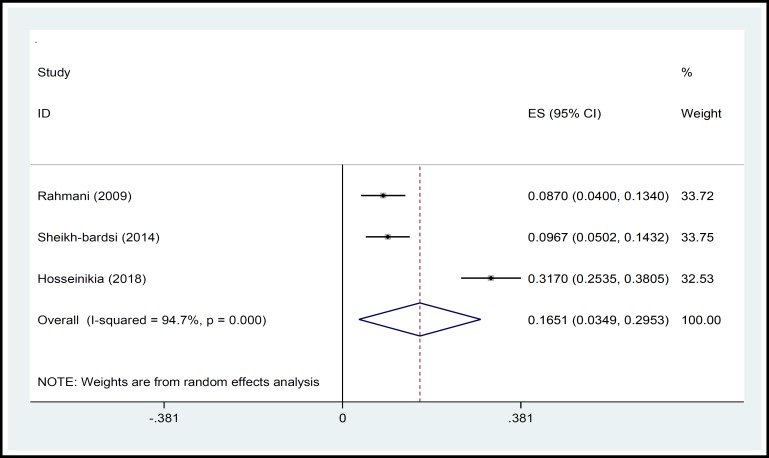
Forest Plot Shows the Prevalence Rate of Cultural WPV in Overall and Separately

**Figure 5:**
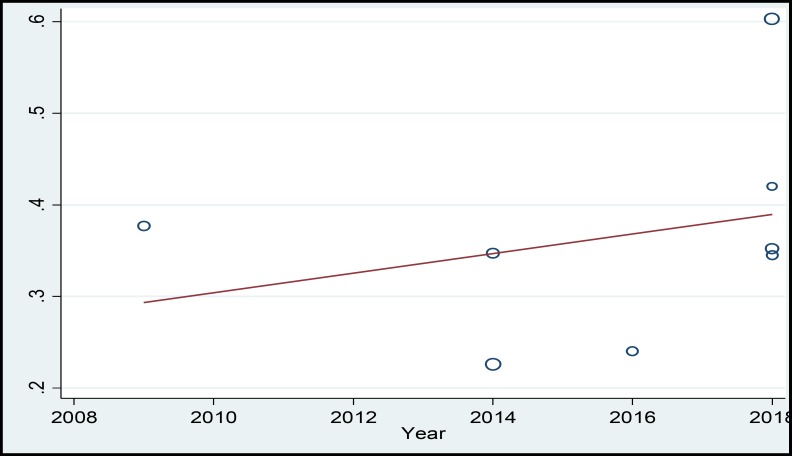
MetaRegression Based on the Relationship between the Study Year and Prevalence of Physical WPV

**Figure 6:**
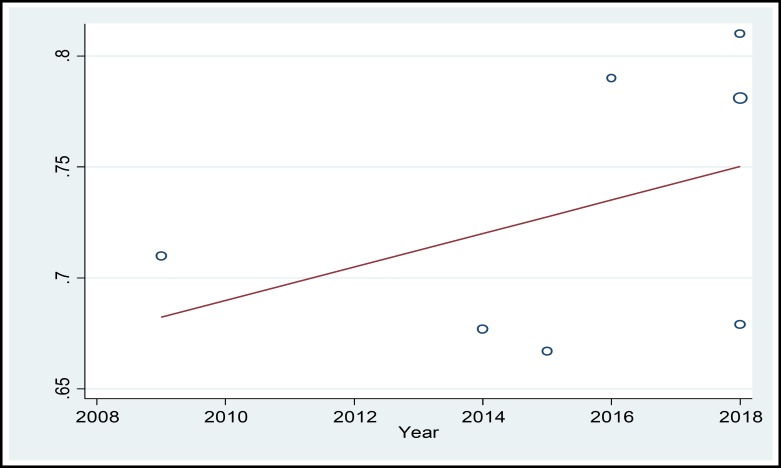
MetaRegression Based on the Relationship between the Study Year and Prevalence of Verbal WPV

**Figure 7:**
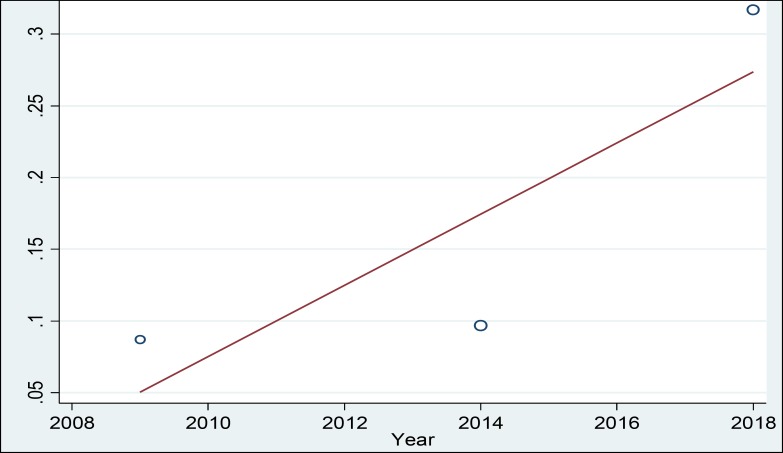
MetaRegression Based on the Relationship between the Study Year and Prevalence of Cultural WPV

**Figure 8:**
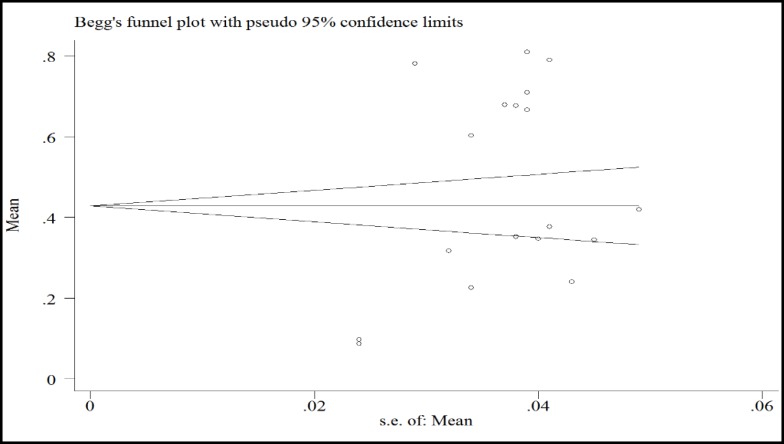
Begg’s Funnel Plot for Publication Bias

In a systematic review, 164 relevant studies were identified. After title and abstract screening, 150 studies were excluded. Then, the full-text of 14 studies were assessed for eligibility. Finally, 9 qualified studies entered the meta-analysis (Figure 1). The total sample size in the study was 1257 men, with an average age of 32.21 ± 2.01years. Sampling method in most of the studies was simple random method and study design was cross sectional in all studies (Table 1). The findings showed that the overall prevalence of physical violence was 36.39% (CI 95%:27.29–45.50, P<0.001, I^2^= 90.8%). The highest prevalence of physical violence was reported in the study by Husseini Kia in Fars, Bushehr, and Kohgiluyeh and Boyer Ahmad provinces as 60.3% (95% CI = 53.62-66.98) and the lowest in in the study by Sheikh-Bardsiri in Kerman province as 22.58% (95% CI = 16-29.16) (Figure 2). The overall prevalence of verbal violence was 73.13% (95% CI = 68.64-77.62, P = 0.013, I^2^= 62.7%), and the highest prevalence rate was reported in the study by Afkhamzadeh in Sanandaj as 81% (95% CI=73.31-88.69) and the lowest in the study by Maghami in north of Khuzestan province as 66.7% (95%CI=59-74.40) (Figure 3). The overall prevalence of cultural violence was reported as 16.51% (95% CI= 3.49-29.53, p<0.001. I^2^=94.7%) and the highest prevalence was reported in the study by Hosseini Kia in Fars, Bushehr and Kohgiluyeh and Boyer Ahmad provinces as 31.7% (95% CI=25.35-38.05) and the lowest in the study by Rahmani in East Azarbayejan province as 8.7% (95%CI=4-13.40) (Figure 4). The results of meta-regression showed that the prevalence rate of physical and verbal WPV against Iranian EMS personnel has significantly increased with the increase in the study year (Figures 5, 6, and 7). Moreover, the result of Begg’s test revealed that the effect of publication bias was not significant (p = 0.361) (Figure 8). 

## Discussion

A review of the studies revealed that the prevalence of types of WPV in various studies has been reported differently, which could be due to the lack of a single measurement tool and different measurement methods. The results of this study showed that the prevalence of physical, verbal, and cultural WPV against EMS personnel in Iran was 36.39% (CI 95%: 27.29 – 45.50, P < 0.001, I^2^ = 90.8%), 73.13% (95% CI = 68.64-77.62, P = 0.013, I^2^ = 62.7%), and 16.51% (95% CI =3.49- 29.53, p<0.001. I^2^=94.7%), respectively. According to the study by Bigham in the United States, verbal and physical WPV in prehospital emergency staff was 67% and 26%, respectively (27). In the study by Kaeser in Switzerland, physical and verbal WPV were reported as 56% and 92%, respectively (8). Therefore, in the present study, similar to studies conducted in other countries, verbal violence has a high prevalence rate. The high prevalence of verbal violence can be due to stress and concern of the patients’ family who try to gain the attention of the staff to their patients to receive more services by threatening, humiliating, and using violence against EMS personnel. The results of the meta-regression showed that the prevalence rate of the physical, verbal, and cultural WPV against Iranian EMS personnel was increasing by the increase in the study year (Figures 5, 6, and 7) which could be due to the larger use of prehospital emergency services by people and the widespread availability of these services. As the number of services increases, the number of violence cases increases as well. Other reasons that can increase the prevalence of WPV include the concern of the patients and their families as well as the family’s fear of patient's death due to inappropriate and delayed medical care and delayed transfer of the patient to the nearest medical center. Studies conducted in other countries showed that factors such as illiteracy and insufficient education of patients’ companions, lack of protocols to control WPV, delay in the presence of emergency medical technicians on the scene, drug abuse, alcohol consumption, mental disorders, unexpected diseases, damage or sudden death, absence of security staff on the scene, and people’s unawareness of EMS duties are the major causes of workplace violence against EMS personnel (23-26 and 28). Comparison of WPV in different regions of Iran shows that the highest prevalence of physical, verbal, and cultural WPV are found in south of the country and the lowest in northwest of the country. Differences in beliefs, attitudes, traditions, culture, and climatic conditions are the factors that can be effective in prevalence and expression of violence (29). In the study by Rahmani, 67% of the participants noted that people’s unawareness of the duties of the emergency medical personnel was one of the main causes of WPV, and about 96% of the participants reported that they needed training in anger and stress management. This finding shows that anger management training can be one of the best ways to reduce WPV against medical emergency staff. The training can be conducted at different levels. The first level is training the public about performance and duties of prehospital emergency staff, and the second level is to provide training on stress and violence management to prehospital emergency personnel. Due to the unique occupational conditions of EMS, full control of violence in prehospital emergency is very difficult, but developing the skills to identify and control violence is highly important. The violence between prehospital emergency staff and patients and their families destroys the mutual trust, which is a prerequisite for providing medical care and may lead to unpleasant outcomes. Many people are unaware of the tasks of EMS personnel and see them as physicians in many cases and expect them to promptly treat and prescribe medication, and if they do not meet this expectation, the patients or their companion may behave violently. According to the studies, another major cause of violence in prehospital emergency services is delay in the arrival of the ambulance to the accident site. According to the current standards, in cities, the arrival time of ambulance to the accident site is 3 to 5 minutes (30-33). Despite many advances in our country’s prehospital emergency services, it is impossible to meet the standard time and patients and their families and people who are at the scene, especially in the case of out-of-home events, blame the medical personnel, which leads to violent behavior against EMS personnel. The results of the systematic review and meta-analysis by Dalvand (34) showed that the prevalence of physical and verbal violence against nurses in Iran was 28% and 74%, respectively. 

The square sign shows the prevalence rate of physical WPV for each study. Diamond sign at the bottom of the figure shows the overall prevalence rate of physical WPV for all the studies. The diamond diameter also shows the 95 % confidence interval of the overall prevalence of physical WPV.

The square sign shows the prevalence rate of verbal WPV for each study. Diamond sign at the bottom of the figure shows the overall prevalence rate of verbal WPV for all the studies. The diamond diameter also shows the 95 % confidence interval of the overall prevalence of verbal WPV.

The square sign shows the prevalence rate of cultural WPV for each study. Diamond sign at the bottom of the figure shows the overall prevalence rate of cultural WPV for all the studies. The diamond diameter also shows the 95 % confidence interval of the overall prevalence of cultural WPV.

**Table 1:** The Characteristics of the Selected Studies in the Meta-Analysis

**First author**	**Place**	**Year**	**Sample** **size**	**Average ** **age**	**Physical ** **violence**	**Verbal ** **violence**	**Cultural ** **violence**	**STROBE ** **score**	**Sampling ** **method**
Maghami(18)	North of Khuzestan	2014	144	32.9	34.7%			32	Random sampling
Maghami(19)	North of Khuzestan	2015	144	32.9		66.7%		32	Random sampling
Sheikh-bardsiri(20)	Kerman	2014	155	28	22.58%	67.7%	9.67%	30	Random sampling
Hosseinikia(21)	Fars, Bushehr, and Kohgiluyeh and Boyer-Ahmad	2018	206	31.77	60.3%	78.1%	31.7%	37	Random sampling
Rahmani(22)	East Azerbaijan	2009	138	33.76	37.7%	71%	8.7%	36	Random sampling
Bozorgi(23)	Mazandaran	2018	157	32.15	35.2%	67.9%		29	Random sampling
Soheili(24)	Urmia	2016	100	31.6	24%	79%		37	Random sampling
Afkhamzadeh(25)	Sanandaj	2018	100		42%	81%		36	Census
Golzare (26)	Gilan	2018	113	37.2	34.51%			34	Random sampling

## Limitation

The number of studies on WPV against EMS personnel in Iran was limited and 2 out of 3 studies were only focused on physical violence. In addition, verbal violence was investigated only in one study. In neither of the studies, the risk factors of WPV, such as alcohol consumption and psychiatric problems in patients and their companions, were studied. Another limitation was high heterogeneity in the results.

## Conclusion

WPV against EMS personnel is one of the problems in the health system that is increasingly growing worldwide and has widespread effects that can lead to termination of employment and job dissatisfaction. Therefore, further studies should be conducted to identify the underlying causes of this type of violence. Moreover, EMS personnel should be provided with training on violence prevention methods and how to deal with violence of the patients and their companions. Also, adopting supportive policies, such as simultaneous presence of police with EMS on the scene of accidents, legal prosecution of abusers, culture building, and raising public awareness about the critical role of EMS personnel in the health system, and promotion of their occupational position, are some strategies proposed to reduce violence against EMS personnel.
